# Perfusate Biomarker Comparison During Renal Hypothermic and Normothermic Machine Perfusion: Do These Techniques Provide Similar Insights?

**DOI:** 10.1097/TP.0000000000005440

**Published:** 2025-06-09

**Authors:** Tim L. Hamelink, Baran Ogurlu, Chris L. Jaynes, Veerle A. Lantinga, Henri G.D. Leuvenink, Anna K. Keller, Cyril Moers

**Affiliations:** 1 Department of Surgery–Organ Donation and Transplantation, University Medical Center Groningen, University of Groningen, Groningen, the Netherlands.; 2 34Lives, Public Benefit Corporation, West Lafayette, IN.; 3 Department of Urology, Aarhus University Hospital, Aarhus, Denmark.; 4 Department of Clinical Medicine, Aarhus University, Aarhus, Denmark.

## Abstract

**Background.:**

Hypothermic machine perfusion (HMP) and normothermic machine perfusion (NMP) are increasingly used in renal transplantation. Both techniques enable pretransplant organ viability assessment through biomarker measurements in the perfusion solution. This study examines similarities and differences in biomarker release during HMP and NMP, focusing on well-established biomarkers alongside functional markers in porcine and discarded human donor kidneys.

**Methods.:**

Discarded human donor kidneys (n = 25) underwent 4 h of oxygenated hypothermic machine perfusion (HMPO_2_) and subsequently 4 h of NMP. Porcine kidneys were exposed to either minimal warm ischemia or 75 min of warm ischemia (n = 30 per group). Hereafter, kidneys were placed on HMPO_2_ for 6 h followed by 6 h of NMP. Flow dynamics were recorded, and the biomarkers aspartate aminotransferase (ASAT), lactate dehydrogenase (LDH), *N*-acetyl-β-glucosaminidase, tissue inhibitor of metalloproteinases-2 (TIMP-2), and heart-type fatty acid–binding protein were measured longitudinally in the perfusates.

**Results.:**

For human kidneys, we found moderate to strong correlations between ASAT, LDH, TIMP-2, and heart-type fatty acid–binding protein content measured during HMPO_2_ and the same biomarkers during NMP. In porcine kidneys, clear distinctions between ischemically damaged and healthy kidneys were observed in flow dynamics and content of ASAT, LDH, and TIMP-2 during both HMPO_2_ and NMP.

**Conclusions.:**

Our findings suggest that biomarker release during HMPO_2_ and NMP have similarities, indicating that some biomarkers might already be assessed during HMPO_2_. However, the predictive value of biomarkers in both techniques remains elusive. Additionally, NMP could provide important benefits over HMPO_2_, including functional assessment and reconditioning.

## Graphical abstract

**Figure:**
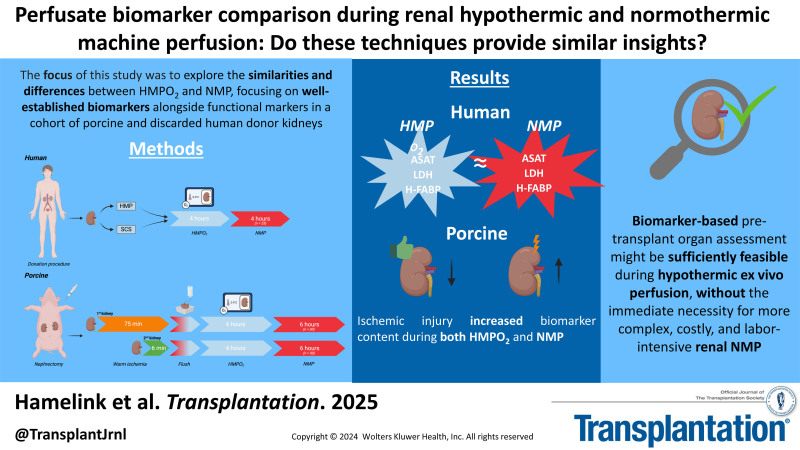

## INTRODUCTION

The donation process of kidneys from deceased donors subjects the organs to nonphysiological stressors that affect viability. Hypothermic machine perfusion (HMP) is being adopted as the standard of care in several countries to optimize organ preservation after retrieval. This technique has a better ability to conserve deceased-donor kidney quality than static cold storage.^1,2^ Besides being a superior preservation technique, HMP also provides a window of opportunity to perform pretransplant organ viability assessment. Indeed, it has been shown that renal vascular resistance (RR) during cold perfusion is an independent risk factor for delayed graft function (DGF); however, its predictive accuracy is very poor.^3,4^ Several perfusate biomarkers during HMP have also been investigated. One clinical study showed that glutathione-S-transferase, heart-type fatty acid–binding protein (H-FABP), and *N*-acetyl-β-glucosaminidase (NAG) in the HMP perfusate were independently associated with DGF but not with graft survival.^5^ Normothermic machine perfusion (NMP) is often believed to provide an even better platform than HMP, allowing optimized preservation, pretransplant viability assessment, and resuscitation, because the organ resumes metabolic activity when it is warmed up.^6,7^ Despite the fact that NMP is already in use for pretransplant assessment of livers, lungs, and hearts, its added value in terms of predicting outcomes of kidney transplantation remains to be proven. A variety of conventional functional markers (eg, urine production, creatinine clearance, fractional sodium excretion) alongside a range of perfusate biomarkers have been examined in preclinical studies.^8-12^ However, NMP has several disadvantages, such as the extension of preservation times, higher costs, and an increased risk of damaging the graft in case of technical issues. Although the added value of biomarker-based organ assessment during both HMP and NMP is still being studied, we also need to gain a deeper understanding of how biomarker release is similar or differs between these techniques. Thus, the optimal timing and perfusion method for biomarker-based viability assessment can be determined. Our study explored the similarities and differences between HMP and NMP, focusing on well-established perfusate biomarkers alongside conventional functional markers in porcine and discarded human donor kidneys.

## MATERIALS AND METHODS

### Study Design and Ethical Approval

**FIGURE 1.**
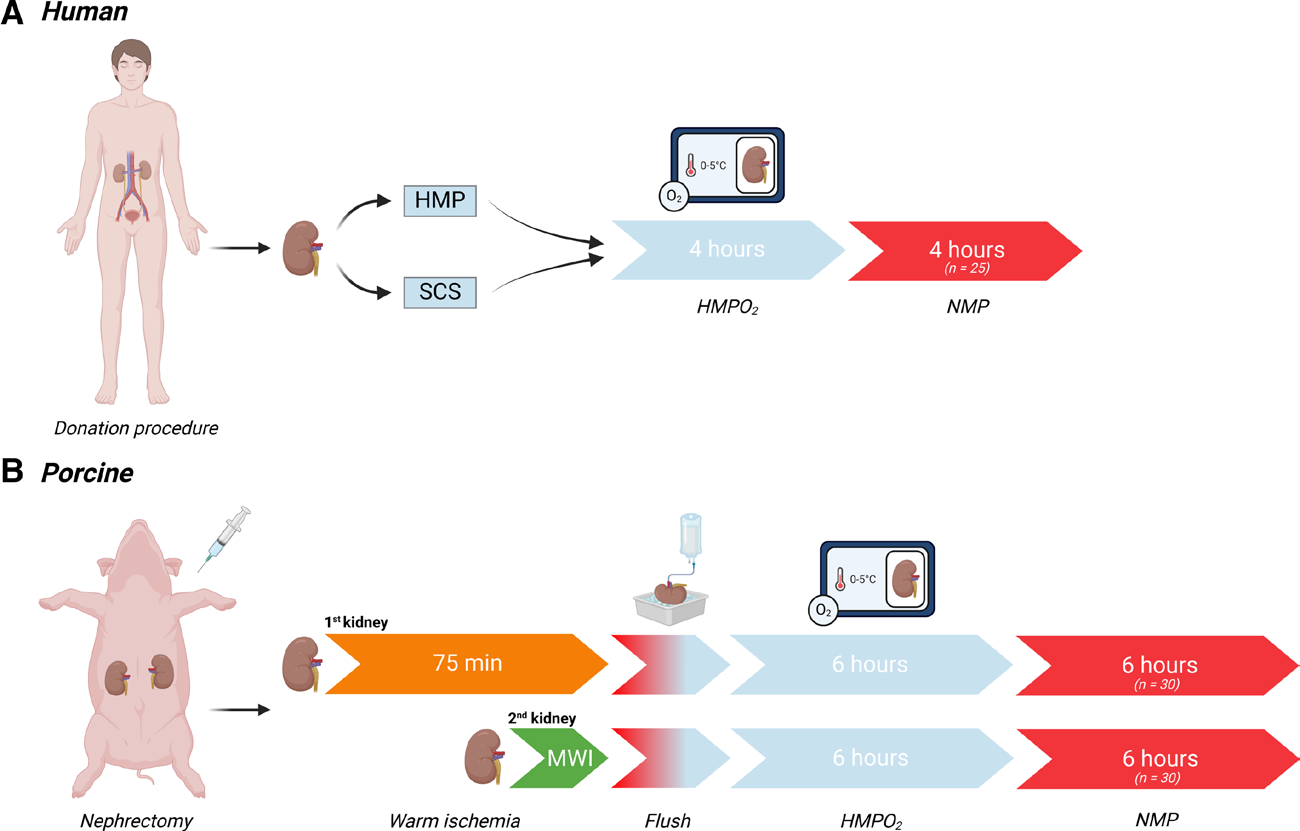
Study design. Human deceased-donor kidneys, deemed unsuitable for transplantation, were preserved with either SCS or with HMP on arrival at our laboratory (A). Kidneys were placed on HMPO_2_ and perfused for 4 h. Subsequently, kidneys were connected to an NMP device and perfused for another 6 h. To ensure a fair comparison, only the first 4 h of NMP were used in this study, aligning it with the total HMPO_2_ duration. Porcine kidneys were randomized to sustain a MWI, or 75 min of WI (B). After bilateral nephrectomy, both kidneys were flushed with cold preservation solution. Subsequently, kidneys were connected to an HMPO_2_ device and perfused for 6 h. Hereafter, kidneys were connected to an NMP circuit and perfused for an additional 6 h. HMP, hypothermic machine perfusion; HMPO_2_, oxygenated HMP; MWI, minimal period of warm ischemia; NMP, normothermic machine perfusion; SCS, static cold storage.

In West Lafayette, United States, human deceased-donor kidneys (n = 25), deemed unsuitable for transplantation, were included with consent for use of the kidneys for research according to the Uniform Anatomical Gift Act in the United States. Kidneys were initially preserved with static cold storage or HMP on arrival at our laboratory and were subsequently connected to an oxygenated HMP (HMPO_2_) device (Figure 1A). After 4 h of HMPO_2_, the kidneys were connected to an NMP setup and perfused for 6 h. To ensure a fair comparison between both perfusion techniques, we chose to only use the first 4 h of NMP, aligning it with the 4-h HMPO_2_ duration.

In Aarhus, Denmark, porcine kidneys (n = 60) were retrieved from 30 anesthetized female laboratory pigs (Figure 1B). From each kidney pair, 1 organ was randomized to sustaining a minimal period (6 min) of warm ischemia (WI), and the other kidney underwent 75 min of WI. After organ retrieval, both kidneys were placed on HMPO_2_ for 6 h. Subsequently, both kidneys were exposed to 6 h of NMP. Animal care and surgical procedures adhered to regulations set by local authorities and the European Union (directive 2010/63/EU), and approval was received from the Animal Experimentation Council in Denmark (reference No. 2020-15-0201-00624) and the European Research Council Ethics Committee.

### Oxygenated HMP

Discarded human kidneys were cannulated with either a 5-mm straight cannula or a SealRing cannula (also Organ Recovery Systems), depending on the vascular anatomy of the kidney. Hereafter, kidneys were placed on a custom-built HMPO_2_ device, that was primed with 330 mL of University of Wisconsin Machine Perfusion solution (Belzer UW-MP solution, Bridge to Life Ltd, Columbia, SC), and perfused at a mean arterial pressure of 25 mm Hg for 4 h. Porcine kidneys were cannulated with a 5-mm straight cannula (Organ Recovery Systems, Itasca, IL) and flushed with 300 mL of 4 °C University of Wisconsin cold storage solution (Belzer UW-CS solution, also Bridge to Life Ltd) at a hydrostatic pressure of 100 cmH_2_O. Next, kidneys were placed on an HMPO_2_ device and perfused at a mean arterial pressure of 25 mm Hg for 6 h. The perfusion solution of both the discarded human and porcine HMPO_2_ was oxygenated with 100% oxygen at a rate of 0.1 L/min. Continuous measurements of perfusate flow were conducted during discarded human and porcine kidney HMPO_2_.

### Normothermic Machine Perfusion

After the HMPO_2_ period, all kidneys were placed on NMP. The perfusion setups were comparable with previously described methods by our group.^8,13,14^ For the human experiments, the perfusion circuit was primed with a perfusion solution based on human O-negative or positive red blood cells, whereas the porcine kidneys were perfused with an autologous porcine red blood cell–based perfusate. The perfusate was supplemented with albumin, crystalloids, creatinine, antibiotics, and electrolytes (**Table S1, SDC,** https://links.lww.com/TP/D278). Pulsatile arterial pressure was set at 110/70 mm Hg and the perfusate was oxygenated with carbogen (95% O_2_/5% CO_2_) at a rate of 0.5 L/min. Total renal blood flow (RBF) was continuously measured. In the discarded human renal NMP, a calcium antagonist was continuously infused (**Table S1, SDC,** https://links.lww.com/TP/D278). In addition, if glucose concentration reduced to <4.0 mmol/L, a continuous infusion of 3 mL/h glucose was started and a bolus of glucose 5% was administered to the perfusate to reach a circulating glucose concentration of 6 mmol/L. During the porcine perfusions, the circuit was continuously supplemented with a mixture of a calcium antagonist, amino acids, insulin, glucose, and vitamins.

### Sample Collection and Biochemical Analyses

A detailed description of the sampling procedures and biochemical analyses can be found in **Supplemental Materials and Methods** (**SDC,** https://links.lww.com/TP/D278).

### Statistical Analyses

Statistical analyses and data visualizations were performed using GraphPad Prism (version 9.3.1, GraphPad Software Inc, La Jolla, CA). Q-Q plots were composed to analyze the distribution of the data. For normally distributed continuous data, means with SDs were computed, and comparative analyses were performed using a paired or unpaired *t* test. When continuous data showed a non-Gaussian distribution, medians with interquartile range (IQR) were calculated, and comparisons for unpaired data were made using a Mann-Whitney *U* test, and a Wilcoxon matched-pair signed-rank test was used for paired data. Correlations were calculated using a nonparametric Spearman rank test to evaluate the association of biomarkers during HMPO_2_ and NMP. All *P* values were corrected for multiple comparisons using the Benjamini-Hochberg procedure, and an adjusted 2-sided *P* value of ≤0.05 was considered to indicate statistical significance.^15^

## RESULTS

### Discarded Human Kidneys

#### Flow, Resistance, and Biomarkers During HMPO_2_ and NMP

**FIGURE 2.**
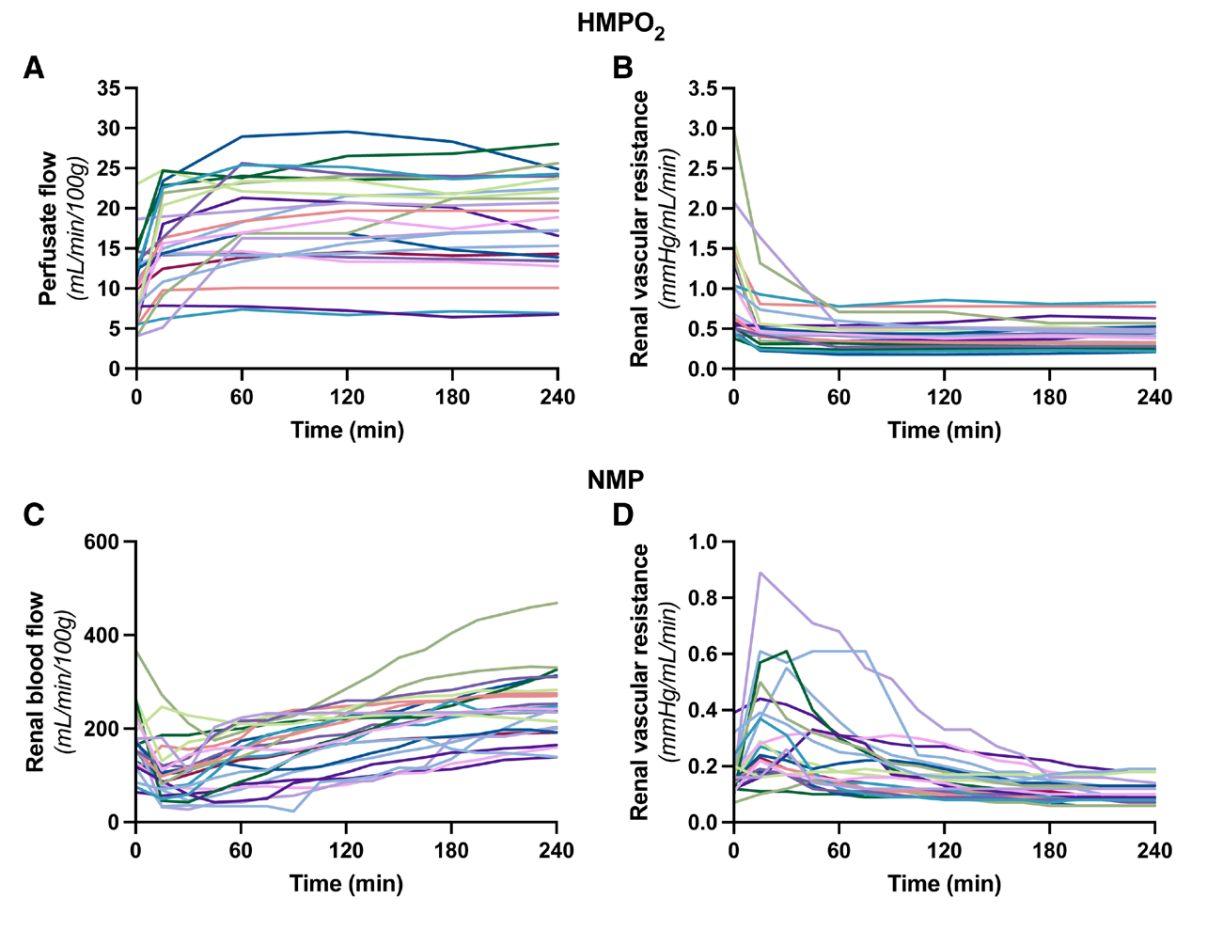
Hemodynamic alterations throughout ex vivo perfusion of discarded human kidneys. Considerable interindividual variability in perfusate flow and renal vascular resistance during both HMPO_2_ (A and B) and NMP (C and D). Each line represents an individual kidney throughout the perfusion period (n = 25). HMPO_2_, oxygenated hypothermic machine perfusion; NMP, normothermic machine perfusion.

Due to considerable variability in baseline donor characteristics of the kidneys (Table 1), a wide range of perfusate flows was observed during HMPO_2_ (Figure 2A). Perfusate flow increased in all discarded human kidneys during the first 15–60 min of perfusion, whereafter it stabilized. This pattern was inversely reflected in the RR, which displayed a decrease throughout HMPO_2_ (Figure 2B). In contrast to perfusate flow during HMPO_2_, the total RBF during NMP decreased in the first 15–30 min of perfusion, followed by an increase for the remainder of the perfusion (Figure 2C). Conversely, the measured RR increased briefly during the first 15–30 min of perfusion, and then decreased (Figure 2D).

**TABLE 1.** Donor characteristics

	Age, y	Sex	BMI, kg/m^2^	Donor type	WIT, min	Last serum creatinine, mmol/L	CIT,^*a*^ h:min	KDPI	Glomerulosclerosis score, %	Reason for discard
	60 (48–66)		28 (23.2–36.4)			156 (78–370)	29.5 (25.4–37.1)	88 (65.5–94.5)	10 (0–15)	
K1	60	Female	43.3	DCD	7	47	31:02	90	0	High KDPI
K2	35	Male	26.5	DBD	NA	690	19:38	54	0	Poor function in donor
K3	45	Male	40.4	DBD	NA	359	28:29	89	20	High KDPI
K4	68	Female	25.2	DBD	NA	55	27:42	88	15	High KDPI
K5	60	Male	26.4	DCD	10	644	28:51	95	10	Poor function in donor
K6	52	Male	31	DBD	NA	548	33:35	82	6	Poor function in donor
K7	54	Female	38.6	DCD	11	69	45:09	84	68	Biopsy findings
K8	75	Female	18.9	DBD	NA	80	13:50	98	^*b*^	High KDPI
K9	72	Female	36.5	DBD	NA	76	23:04	95	12	High KDPI
K10	65	Male	28	DCD	8	115	24:15	94	13	High KDPI
K11	67	Male	22.2	DBD	NA	97	14:31	97	46	Biopsy findings
K12	53	Female	23.8	DCD	13	147	29:47	77	^*b*^	Long WIT
K13	69	Male	33.6	DBD	NA	557	31:57	89	0	High KDPI
K14	47	Male	30	DBD	NA	306	37:03	78	20	Poor function in donor
K15	58	Male	31	DBD	NA	156	28:39	87	0	^*b*^
K16	49	Female	20.5	DBD	NA	159	29:49	54	35	Biopsy findings
K17	66	Male	37.9	DCD	14	68	32:07	95	0	High KDPI
K18	32	Male	42	DBD	NA	310	26:43	20	0	Biopsy findings (ATN)
K19	61	Female	22.5	DCD	19	43	42:51	21	6	High RR during HMP
K20	60	Male	22.3	DCD	11	87	37:12	89	10	Long functional WIT
K21	64	Male	33.4	DBD	NA	203	43:51	93	13	High KDPI
K22	60	Female	26.2	DBD	NA	235	39:36	85	6	Biopsy findings
K23	65	Male	27.1	DBD	NA	690	24:28	97	12	High KDPI
K24	44	Male	36.2	DCD	10	380	34:40	50	10	Long CIT
K25	36	Female	21.8	DCD	6	108	49:20	38	4	Long CIT

Data are represented as median and interquartile range.

a Time from donor nephrectomy until the start of normothermic machine perfusion.

b Missing

ATN, acute tubular necrosis; BMI, body mass index; CIT, cold ischemia time; DBD, donation after brain death; DCD, donation after circulatory death; HMP, hypothermic machine perfusion; KDPI, kidney donor profile index; RR, renal vascular resistance; WIT, warm ischemia time.

**FIGURE 3.**
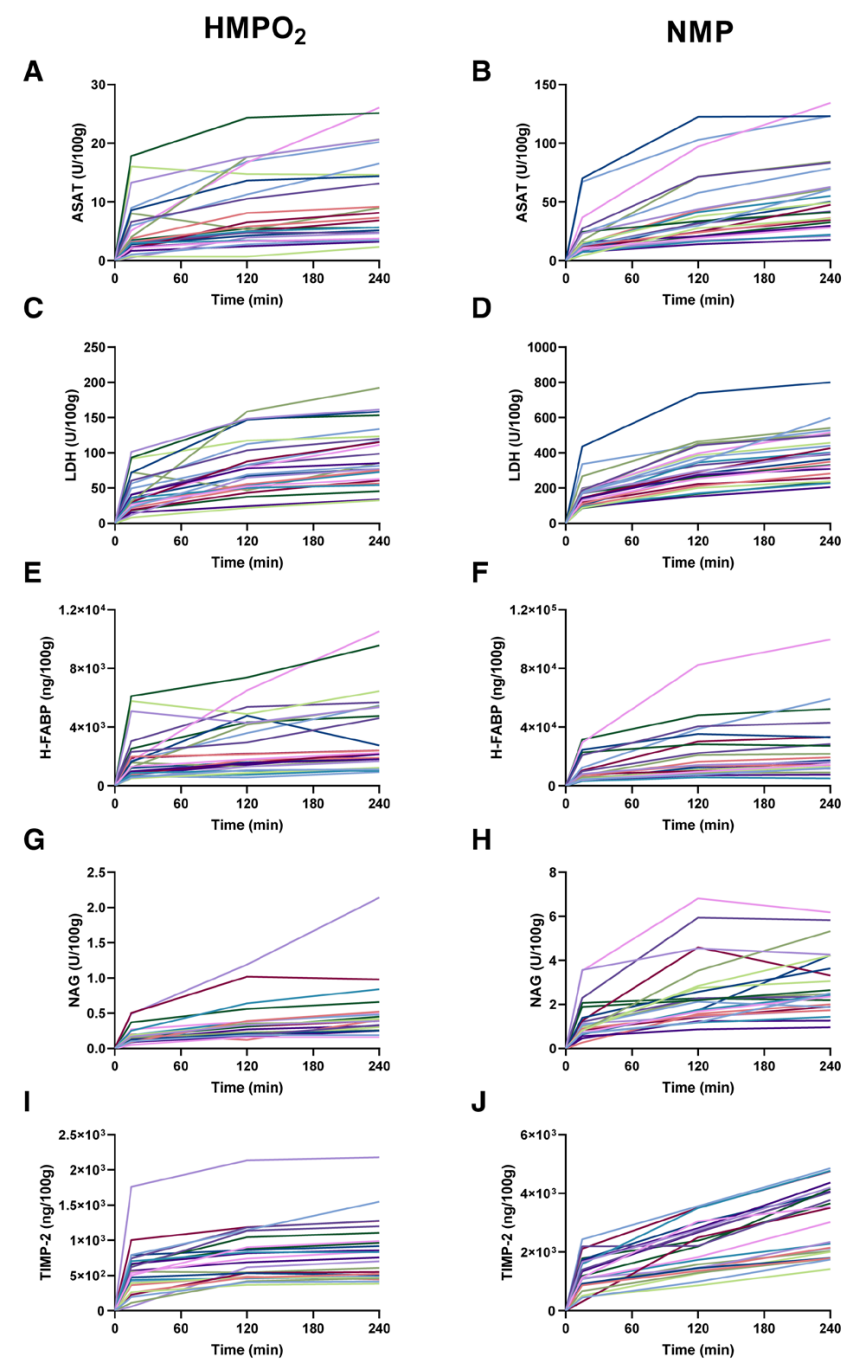
Biomarkers during ex vivo perfusion of discarded human kidneys. Absolute ASAT content during HMPO_2_ (A) and NMP (B). Absolute LDH content during HMPO_2_ (C) and NMP (D). Absolute H-FABP content during HMPO_2_ (E) and NMP (F). Absolute NAG content during HMPO_2_ (G) and NMP (H). Absolute TIMP-2 content during HMPO_2_ (I) and NMP (J). Each line represents an individual kidney throughout the perfusion period (n = 25). ASAT, aspartate aminotransferase; H-FABP, heart-type fatty acid binding protein; HMPO_2_, oxygenated hypothermic machine perfusion; LDH, lactate dehydrogenase; NAG, *N*-acetyl-β-glucosaminidase; NMP, normothermic machine perfusion; TIMP-2, tissue inhibitor of metalloproteinases-2.

The variability observed in the RBF and RR was also reflected in the range of biomarker concentrations measured during HMPO_2_ and NMP. Across all experiments, aspartate aminotransferase (ASAT), lactate dehydrogenase (LDH), H-FABP, NAG, and TIMP-2 increased during both HMPO_2_ and NMP (Figure 3A–J).

#### Association of Biomarkers During HMPO_2_ and NMP

**FIGURE 4.**
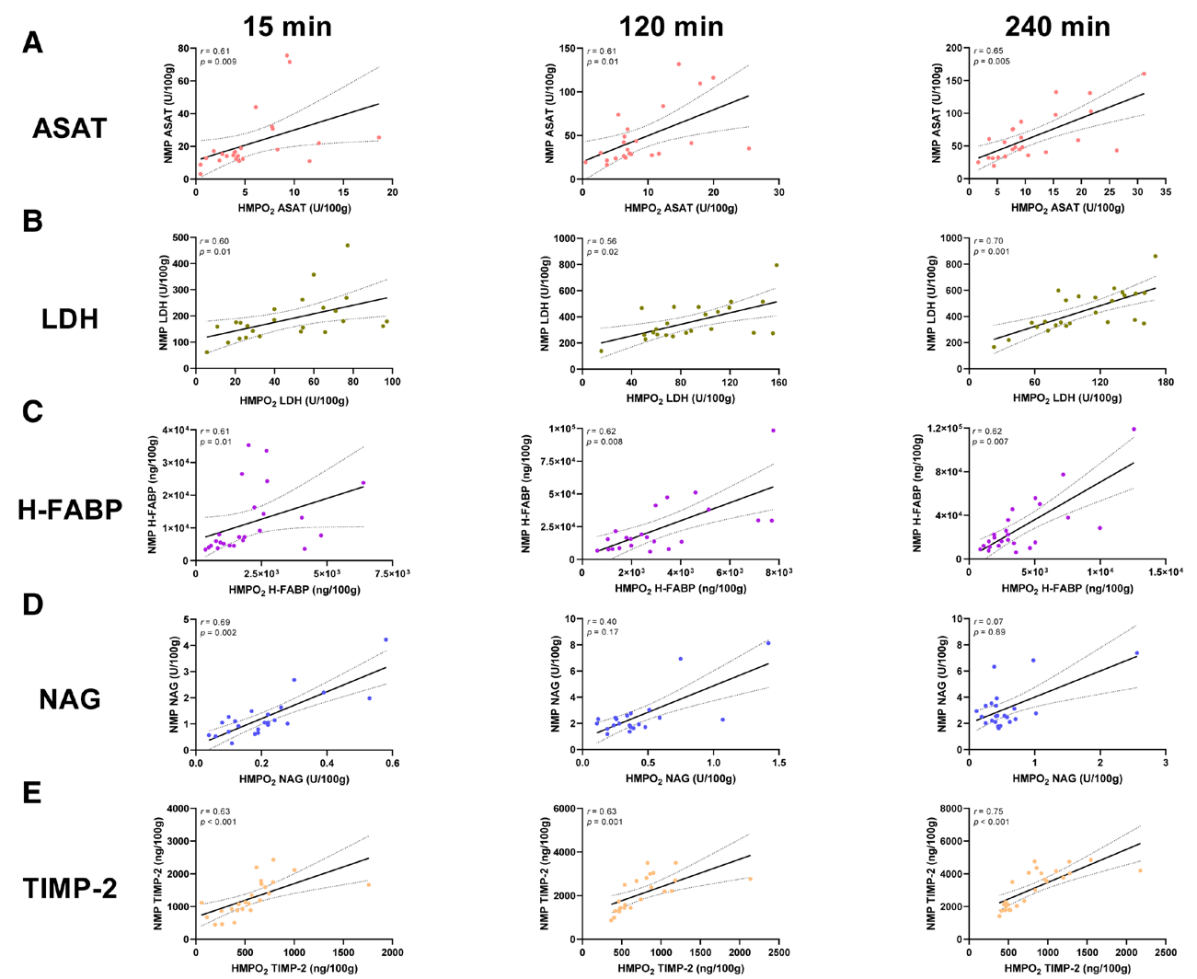
Spearman’s correlation between measured biomarkers during HMPO_2_ and NMP of discarded human kidneys. ASAT (A), LDH (B), H-FABP (C), NAG (D), and TIMP-2 (E) correlations at 15, 120, and 240 min of perfusion. ASAT, aspartate aminotransferase; H-FABP, heart-type fatty acid binding protein; HMPO_2_, oxygenated hypothermic machine perfusion; LDH, lactate dehydrogenase; NAG, *N*-acetyl-β-glucosaminidase; NMP, normothermic machine perfusion; *r*, Spearman correlation coefficient; TIMP-2, tissue inhibitor of metalloproteinases-2.

To illustrate the association between measured biomarkers during HMPO_2_ and NMP at corresponding time points during perfusion, a correlation analysis was conducted. Moderate to strong correlations were noted between ASAT measured during HMPO_2_ and NMP at 15 min (*r* = 0.61, *P* = 0.009), 120 min (*r* = 0.61, *P* = 0.01), and 240 min (*r* = 0.65, *P* = 0.005) of perfusion (Figure 4A). This consistent trend was also observed in LDH measurements, with a moderate to strong correlation across all time points (15 min, *r* = 0.60, *P* = 0.01; 120 min, *r* = 0.56, *P* = 0.02; 240 min, *r* = 0.70, *P* = 0.001; Figure 4B). H-FABP also showed this persistent moderate to strong correlation during all time points of perfusion (15 min, *r* = 0.61, *P* = 0.008; 120 min, *r* = 0.62, *P* = 0.01; 240 min, *r* = 0.62, *P* = 0.007; Figure 4C). A different trend was found for NAG measurements, where a strong correlation was observed at 15 min (*r* = 0.69, *P* = 0.002), but this correlation weakened throughout perfusion (*r* = 0.07, *P* = 0.89; Figure 4D). Moreover, moderate to strong correlations were noted between TIMP-2 measured during HMPO_2_ and NMP at 15 min (*r* = 0.63, *P* < 0.001), 120 min (*r* = 0.63, *P* = 0.001), and 240 min (*r* = 0.75, *P <* 0.001) of perfusion (Figure 4E).

#### Donor Characteristics Correlated With Flow and Biomarker Content During HMPO_2_ and NMP

A correlation analysis was performed to evaluate the association between several key donor characteristics and flow and biomarker measurements. Significant moderate to strong correlations were found between donor serum creatinine and TIMP-2 measured during HMPO_2_ (*r* = 0.751, *P* < 0.001) and NMP (*r* = 0.740, *P* < 0.001; Table 2). However, none of the other analyses reached statistical significance.

**TABLE 2.** Correlation of donor characteristics with functional and biomarker parameters during HMPO_2_ and NMP of discarded human kidneys

Variables	Spearman correlation coefficient	*P*
Donor age		
HMPO_2_ perfusate flow	–0.226	0.47
NMP RBF	–0.018	0.95
HMPO_2_ ASAT	–0.140	0.68
NMP ASAT	0.089	0.82
HMPO_2_ LDH	–0.090	0.81
NMP LDH	0.061	0.88
HMPO_2_ H-FABP	–0.373	0.18
NMP H-FABP	–0.255	0.42
HMPO_2_ NAG	–0.470	0.65
NMP NAG	0.003	0.99
HMPO2 TIMP-2	–0.419	0.06
NMP TIMP-2	–0.265	0.20
Cold ischemia time		
HMPO_2_ perfusate flow	0.308	0.29
NMP RBF	–0.106	0.78
HMPO_2_ ASAT	–0.101	0.79
NMP ASAT	0.052	0.89
HMPO_2_ LDH	–0.328	0.11
NMP LDH	–0.139	0.69
HMPO_2_ H-FABP	–0.176	0.59
NMP H-FABP	0.284	0.35
HMPO_2_ NAG	–0.048	0.90
NMP NAG	–0.293	0.35
HMPO_2_ TIMP-2	0.007	0.98
NMP TIMP-2	–0.032	0.88
Donor serum creatinine		
HMPO_2_ perfusate flow	0.169	0.51
NMP RBF	–0.116	0.76
HMPO_2_ ASAT	0.060	0.88
NMP ASAT	–0.244	0.44
HMPO_2_ LDH	0.045	0.90
NMP LDH	–0.226	0.47
HMPO_2_ H-FABP	0.200	0.51
NMP H-FABP	0.158	0.66
HMPO_2_ NAG	0.498	0.06
NMP NAG	–0.037	0.93
HMPO_2_ TIMP-2	0.751	<0.001
NMP TIMP-2	0.740	<0.001
Donor KDPI		
HMPO_2_ perfusate flow	–0.244	0.45
NMP RBF	0.019	0.96
HMPO_2_ ASAT	–0.237	0.46
NMP ASAT	–0.092	0.82
HMPO_2_ LDH	–0.155	0.81
NMP LDH	0.013	0.88
HMPO_2_ H-FABP	–0.462	0.07
NMP H-FABP	–0.488	0.07
HMPO_2_ NAG	–0.450	0.09
NMP NAG	–0.151	0.68
HMPO_2_ TIMP-2	–0.433	0.09
NMP TIMP-2	–0.210	0.33

ASAT, aspartate aminotransferase; H-FABP, heart-type fatty acid binding protein; HMPO_2_, oxygenated hypothermic machine perfusion; LDH, lactate dehydrogenase; NAG, *N*-acetyl-β-glucosaminidase; NMP, normothermic machine perfusion; TIMP-2, tissue inhibitor of metalloproteinases-2.

**FIGURE 5.**
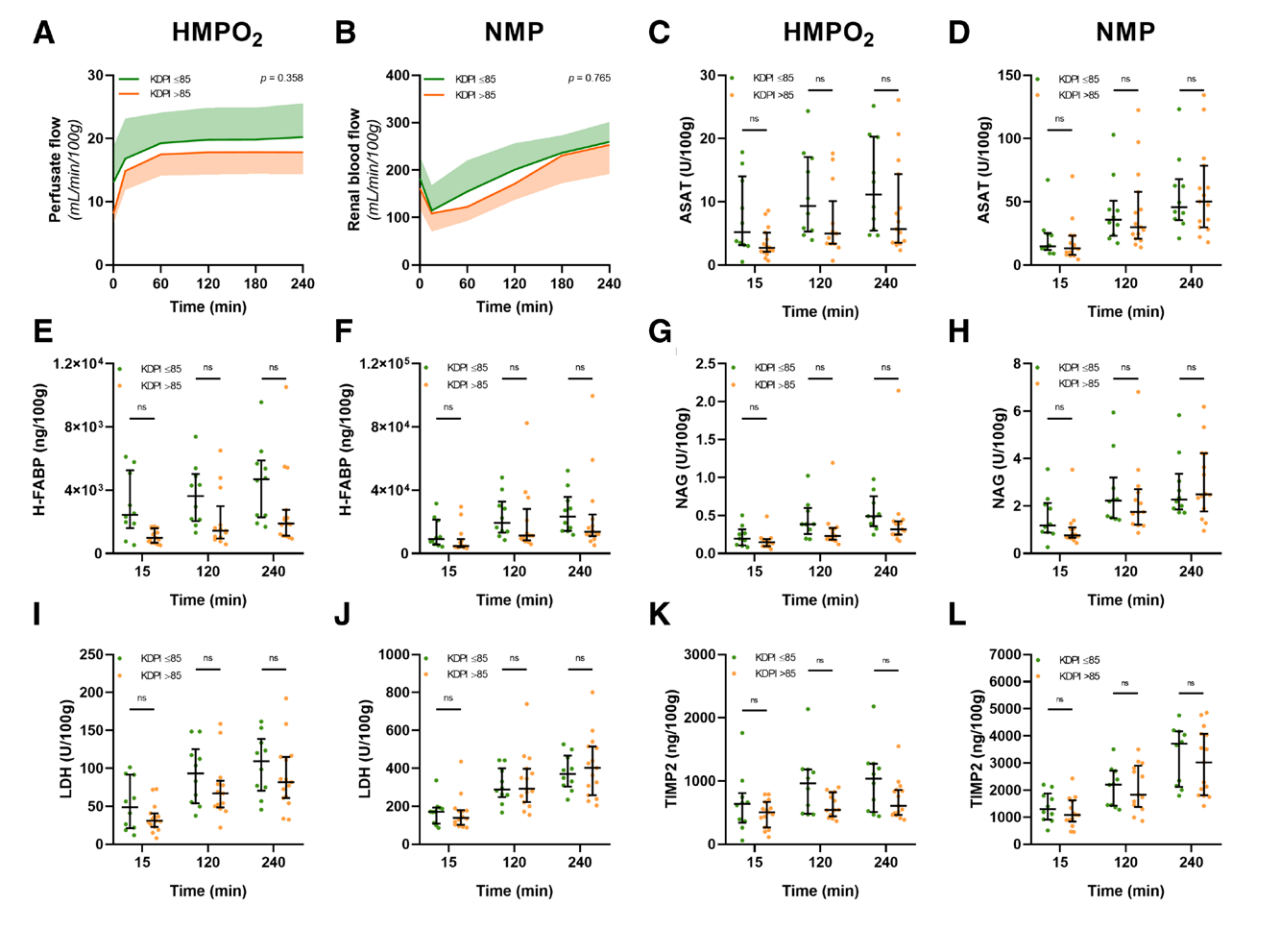
Differences in flow dynamics and biomarkers between human kidneys with a moderate and high KDPI. Arterial flow during HMPO_2_ (A) and NMP (B). Absolute ASAT content during HMPO_2_ (C) and NMP (D). Absolute LDH content during HMPO_2_ (E) and NMP (F). Absolute H-FABP content during HMPO_2_ (G) and NMP (H). Absolute NAG content during HMPO_2_ (I) and NMP (J). Absolute TIMP-2 content during HMPO_2_ (K) and NMP (L). Absolute NAG content during HMPO_2_ (I) and NMP (J). Median and interquartile range are shown. ASAT, aspartate aminotransferase; HMPO_2_, oxygenated hypothermic machine perfusion; KDPI, kidney donor profile index; LDH, lactate dehydrogenase; H-FABP, heart-type fatty acid binding protein; NAG, *N*-acetyl-β-glucosaminidase; NMP, normothermic machine perfusion; ns, not significant; TIMP-2, tissue inhibitor of metalloproteinases-2.

As no significant correlations emerged between the kidney donor profile index (KDPI) as a continuous variable and flow and biomarker measurements, a differentiation was made between kidneys with a high KDPI (n = 13)—defined as >85—and those with moderate KDPI scores (≤85; n = 12), because kidneys with a high score have higher discard rates in the United States.^16^ However, again no significant differences were observed between the moderate and high KDPI groups for all measures during both HMPO_2_ and NMP (Figure 5).

### Porcine Kidneys

#### Flow and Resistance During HMPO_2_ and NMP

**FIGURE 6.**
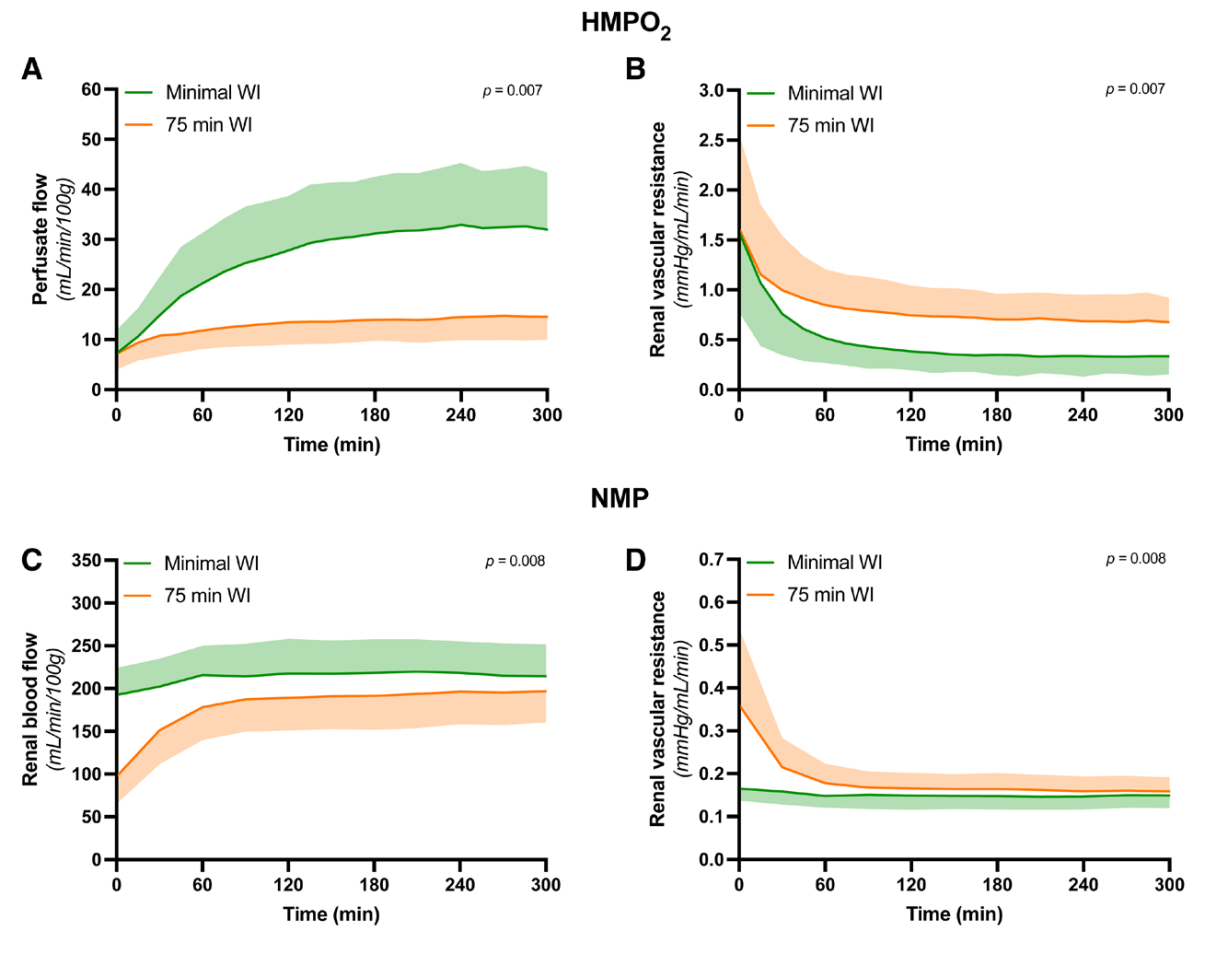
Hemodynamic alterations throughout ex vivo perfusion of porcine kidneys. Arterial flow and renal vascular resistance differences during both HMPO_2_ (A and B) and NMP (C and D) of ischemically and healthy porcine kidneys. Means and SDs are shown. HMPO_2_, oxygenated hypothermic machine perfusion; NMP, normothermic machine perfusion.

During HMPO_2_, perfusate flow clearly increased in the minimal ischemic group in the first 2 h of perfusion, whereafter it stabilized (Figure 6A). In contrast, the 75 min WI group (13.0 ± 4.6 mL/min/100 g) only showed a modest increase in perfusate flow, which remained significantly lower compared with the minimal ischemic group (26.5 ± 12.9 mL/min/100 g, *P* = 0.007). This pattern was inversely observed in the RR (Figure 6B). During NMP, a distinct flow pattern emerged, with total RBF remaining steady throughout perfusion in the minimal ischemic group (213.4 ± 37.2 mL/min/100 g), whereas the flow of the 75 min WI group required approximately 60 min to stabilize (178.8 ± 47.1 mL/min/100 g, *P* = 0.008; Figure 6C). This difference was also reflected in the calculated RR (Figure 6D).

#### Biomarker Content in Ischemically Injured and Healthy Porcine Kidneys During HMPO_2_ and NMP

**FIGURE 7.**
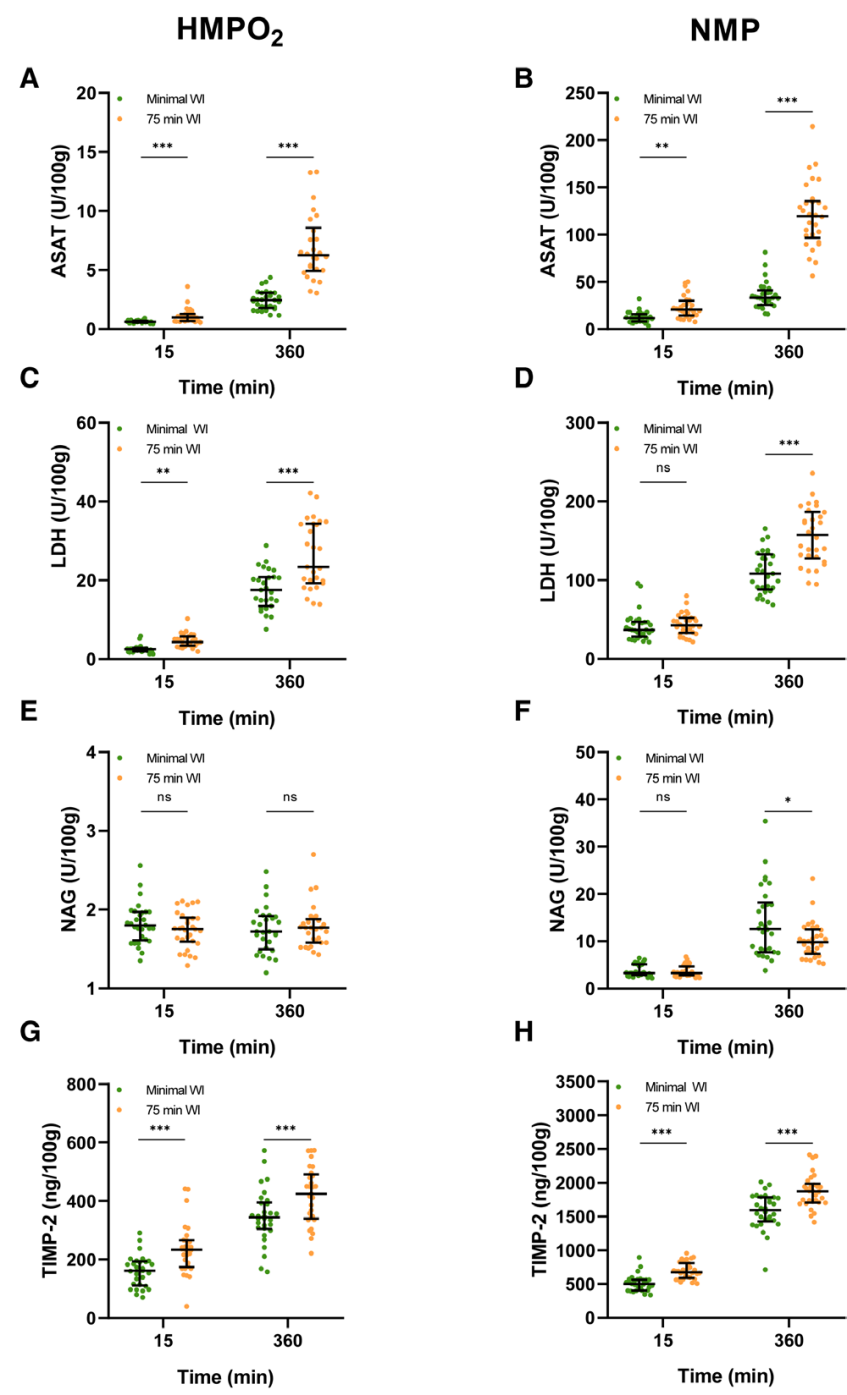
Biomarker differences between ischemically damaged and healthy porcine kidneys during ex vivo perfusion. Absolute ASAT content during HMPO_2_ (A) and NMP (B). Absolute LDH content during HMPO_2_ (C) and NMP (D). Absolute NAG content during HMPO_2_ (E) and NMP (F). Absolute TIMP-2 content during HMPO_2_ (G) and NMP (H). Median and interquartile range are shown. ASAT, aspartate aminotransferase; HMPO_2_, oxygenated hypothermic machine perfusion; LDH, lactate dehydrogenase; NAG, *N*-acetyl-β-glucosaminidase; NMP, normothermic machine perfusion; ns, not significant; TIMP-2, tissue inhibitor of metalloproteinases-2. *P ≤ 0.05, **P < 0.01, ***P < 0.001.

Absolute ASAT content during HMPO_2_ already displayed differences between the minimal WI group and the 75 min WI group (*P* < 0.001) after 15 min of perfusion (Figure 7A and B). This difference was also observed after 15 min of NMP (*P* = 0.001). During 6 h of HMPO_2_ and NMP, biomarker quantity increased in both groups and remained significantly higher in the 75 min WI group compared with the minimal WI group (HMPO_2_, *P* < 0.001; NMP, *P* < 0.001). These differences were also observed in the LDH measurements at both the beginning (*P* = 0.001) and end (*P* < 0.001) of HMPO_2_ (Figure 7C). However, during NMP, this difference was not observed after 15 min of perfusion and only reached significance at the end of perfusion between the minimal WI and 75 min WI group (*P* < 0.001). Absolute NAG content was not significantly different between the 2 groups during the first 15 min of both perfusion techniques and at the end of HMPO_2_ (Figure 7E and F). Surprisingly, at the end of NMP, NAG content was significantly lower in the 75 min group compared with the minimal WI group (*P* = 0.02). Unfortunately, the H-FABP assay was incompatible with our porcine samples, which restricted the quantification of H-FABP content to the human samples only. Absolute TIMP-2 content during HMPO_2_ already displayed differences between the minimal WI group and the 75 min WI group after 15 min HMPO_2_ and the end of perfusion (15 min, *P* < 0.001; 360 min, *P* < 0.001; Figure 7G and H). This difference was also observed during NMP (15 min, *P* < 0.001; 360 min, *P* < 0.001). Median and IQR values can be found in **Table S2** (**SDC,** https://links.lww.com/TP/D278).

#### Area Between the Curves of Ischemically Damaged and Healthy Porcine Kidneys

**FIGURE 8.**
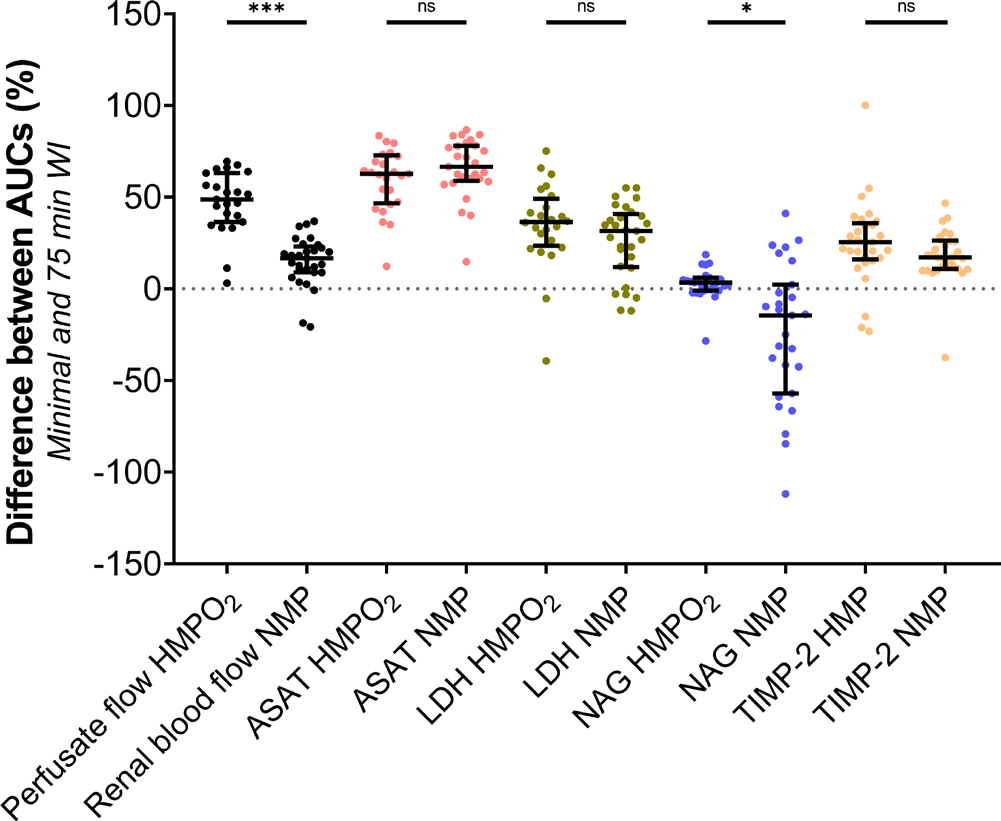
Percentage difference of nonoverlapping AUCs of the minimal WI group and the 75 min WI group of porcine kidneys. Median and interquartile ranges are shown. ASAT, aspartate aminotransferase; AUC, area under the curve; HMPO_2_, oxygenated hypothermic machine perfusion; LDH, lactate dehydrogenase; NAG, *N*-acetyl-β-glucosaminidase; NMP, normothermic machine perfusion; ns, not significant; TIMP-2, tissue inhibitor of metalloproteinases-2; WI, warm ischemia. *P ≤ 0.05, ***P < 0.001.

To determine the perfusion technique during which the most profound differences were observed between the minimal and 75 min WI group, the area under the curve of both the minimal WI and 75 min WI groups was calculated for each biomarker. Subsequently, the percentage overlap between both areas was determined, after which it was subtracted by 100 to obtain the percentage nonoverlapping area (Figure 8). A larger nonoverlapping area, representing the area between the curves, indicates a greater distinction in biomarkers between the minimal and 75 min WI group, thereby revealing during which perfusion technique the most pronounced differences between the groups can be observed.

Perfusate flow during cold perfusion displayed the biggest difference between the 2 groups (48.7% [IQR, 36.5–63.1]) compared with RBF measured during NMP (16.7% [IQR, 7.4–23.1], *P* < 0.001). For ASAT measurements during HMPO_2_, the nonoverlapping area between the 2 groups was 62.8% (IQR, 46.7–72.8), which was comparable with ASAT measured during NMP (66.6% [IQR, 58.9–78]). There were also no differences observed for LDH measured during HMPO_2_ and NMP (HMPO_2_, 36.5% [IQR, 23.4–49.2]; NMP, 31.6% [IQR, 11.8–40.8]). Interestingly, the nonoverlapping area between both groups in NAG content was significantly higher in the minimal WI group (3.4% [IQR, –0.9 to 6]) compared with the 75 min WI group (–14.5% [IQR, –57.1 to 2.3], *P* = 0.01). There were also no differences observed for TIMP-2 measured during HMPO_2_ and NMP (HMPO_2_, 25.5% [IQR, 16.1–35.8]; NMP, 17.1% [IQR, 10.9–26.3]).

## DISCUSSION

This study evaluated a variety of biomarkers and renal flow dynamics during HMPO_2_ and NMP to identify similarities and differences between the 2 techniques. We found that functional markers and biomarkers quantified during both HMPO_2_ and NMP displayed considerable variability in human kidneys, with a moderate to strong correlation between ASAT, LDH, and H-FABP measured during HMPO_2_ and during NMP. In the more controlled circumstances of our porcine model with severe and minimal ischemic injury, significant differences could be detected in terms of flow dynamics and ASAT and LDH during both HMPO_2_ and NMP, with the most notable differences occurring in flow dynamics during HMPO_2_.

Typically, vascular resistance measurements during HMP are considered a valid indicator of graft viability. Indeed, Sandal et al^17^ demonstrated that RR during HMP can be a modest predictor of long-term kidney transplant outcomes. This is in line with a study by Offerni et al^18^ who showed that average flow and resistance, in combination with the KDPI, could predict short- and long-term posttransplant function of kidneys donated after circulatory death. In our porcine cohort, perfusate flow during HMPO_2_ was significantly different between ischemically damaged and healthy grafts. In the context of renal NMP, RBF is also often considered an important quality assessment marker and it is integrated into the ex vivo normothermic perfusion score of the Cambridge group.^19^ Preclinical studies have shown that increasing the duration of WI results in an elevated RR during renal NMP and that baseline RR correlates with posttransplant renal function.^20^ Nevertheless, in a randomized clinical NMP study, Hosgood et al^21^ did not find any predictive value of the above-mentioned assessment score. Flow measurements during porcine renal NMP also revealed a significant difference between the minimal WI and 75 min WI group. Although the disparity in flow measurements between the 2 groups was noticeable in both techniques, the clearest difference was observed during HMPO_2_. These results suggest that measuring flow to distinguish kidneys with acute ischemic injury from undamaged grafts can be done reliably during HMPO_2_, rendering further flow measurements during NMP unnecessary.

In addition to flow dynamics, quantifying graft injury through biomarkers could play an important role in guiding perfusion-based decision-making strategies. However, consensus is lacking on which biomarkers bear the greatest potential to reflect injury or viability during ex vivo perfusion.^22^ The majority of clinical data on biomarkers originate from HMP studies, where, to date, these markers have demonstrated only limited prognostic value.^23^ ASAT and LDH are widely recognized as markers for assessing general cellular injury. In the context of clinical renal HMP, LDH has been associated with DGF and primary nonfunction but not with long-term graft survival, whereas ASAT did not show such predictive potential.^5,24^ A preclinical autotransplantation study suggested that LDH levels do not predict posttransplant renal function, whereas ASAT levels could have predictive value.^20^ In our study, in human kidneys, there was a moderate to strong correlation between ASAT and LDH during HMPO_2_ and the same biomarkers during NMP. This, in conjunction with the significant differences detected between injured and healthy porcine grafts during both HMPO_2_ and NMP, indicates that these specific biomarkers could already be sufficiently indicative of injury when measured during hypothermic perfusion.

Besides biomarkers that assess general cellular damage, markers that provide insight into the site of injury are also commonly quantified in perfusion solutions.^6,23^ H-FABP is a cytoplasmic protein that is present in distal renal tubules. Elevated levels have been associated with ischemic tubular damage and have proven to effectively detect acute kidney injury.^25,26^ Parikh et al^27^ demonstrated that liver-type FABP levels were modestly associated with 6-m posttransplantation glomerular filtration rate. In our analysis, there was a consistently moderate to strong correlation between H-FABP at all measured time points during HMPO_2_ and the same time points during NMP in the human cohort. Although the added value of this specific biomarker has yet to be unraveled, these results show that it could already be sufficiently measured during HMPO_2_. NAG, which is a lysosomal enzyme located in proximal tubular cells, is released in response to ischemic injury and has been independently linked to DGF.^5,28^ However, it did not show an association with long-term outcomes. We found a moderate to strong correlation between HMPO_2_ and NMP NAG content after 15 min of perfusion, which weakened during subsequent measurements. Moreover, NAG failed to reveal the impact of WI during HMPO_2_. These results reflect that NAG might not be an effective marker for assessing acute renal injury during these ex vivo perfusion techniques. Moreover, TIMP-2 content was quantified to assess cell cycle arrest, a marker that has been able to detect acute kidney injury.^29^ TIMP-2 measurements demonstrated a moderate to strong correlation between HMPO_2_ and NMP in human kidneys. In the porcine kidneys, TIMP-2 levels clearly differentiated between ischemically and minimally ischemically injured kidneys across both perfusion techniques. Notably, TIMP-2 was the only marker that showed a strong correlation with donor serum creatinine during HMPO_2_ and NMP in the human cohort, reflecting preexisting donor kidney function. This suggests that TIMP-2 has a unique ability to reflect donor-related impaired kidney function during machine perfusion.

The KDPI is an established scoring system used within the United States to assess the expected functionality of a donor kidney relative to those transplanted in the preceding year. Clinicians use KDPI scores to make informed decisions regarding the suitability of kidneys for transplantation.^30^ Given the propensity for kidneys with higher KDPI scores to be discarded, a division was made between kidneys with a moderate and those with a high KDPI score to explore whether these 2 groups would show a difference in flow dynamics and biomarkers during NMP and HMPO_2_. However, our results revealed no differences in these parameters between kidneys with a moderate and those with a high KDPI score, suggesting that the KDPI score and perfusion-based assessment strategies may each contribute unique perspectives that could provide additive data for organ assessment, rather than one being a surrogate for the other. However, further research that incorporates posttransplant outcome data is required to investigate whether these measurements could indeed reveal different aspects of organ quality.

This study had several limitations. Although several biomarkers that we analyzed were capable of detecting acute injury, we did not explore their capacity to quantify preexisting chronic organ damage. Because such chronic injury also affects posttransplant function and graft longevity, the identification of biomarkers that can detect chronic injury would enhance the relevance of biomarker-based kidney assessment during machine perfusion. Additionally, our study did not incorporate transplantation of porcine and human kidneys and could therefore not establish how injury, measured during HMPO_2_ and NMP, translates into posttransplant function. However, the primary aim of this study was to examine the similarities and differences in biomarker profiles during HMPO_2_ and NMP. Moreover, both models had specific limitations. In the human cohort, the prolonged and variable cold ischemia time (CIT), combined with significant heterogeneity in donor characteristics, resulted in substantial data variability, which limits the strength of the conclusions that can be drawn. In contrast, the primary limitation of the porcine cohort was the relatively short CIT, which does not accurately reflect the longer CIT typically observed in clinical practice.

In conclusion, this study showed a moderate to strong correlation between ASAT, LDH, H-FABP, and TIMP-2 release during HMPO_2_ and the same biomarkers during NMP. We found that ASAT and LDH can already reflect the impact of acute injury during hypothermic perfusion. Moreover, ex vivo perfusate flow showed the greatest capability to differentiate between ischemic and minimally ischemic kidneys during HMPO_2_. Although the prognostic value of functional and biomarker-based organ evaluation during both HMPO_2_ and NMP remains to be elucidated, our findings suggest that certain assessments might already be performed during the clinically more established and safer hypothermic perfusion. However, further investigation is needed to determine whether this approach can sufficiently reduce the need for biomarker-based assessment during renal NMP. Moreover, NMP does offer a broader assessment platform, as it allows for the evaluation of functional markers such as urine production, creatinine clearance, and fractional sodium excretion. Therefore, future studies should focus on combined analyses by incorporating data from both perfusion techniques. This may offer a more comprehensive approach to ex vivo organ evaluation and to further enhance our knowledge about their similarities and differences in predicting posttransplantation outcomes.

## ACKNOWLEDGMENTS

The authors thank the Organ Procurement Organizations (Kentucky Organ Donor Affiliates (Louisville, KY), Gift of Hope (Chicago, IL), Texas Organ Sharing Alliance (San Antonio, TX), LifeLine of Ohio (Columbus, OH), and LifeGift (Houston, TX) for providing organs for research purposes; the donors and their families for their permission to use discarded donor kidneys for research purposes; 34Lives for providing their facilities and resources to conduct human kidney perfusions, in particular Stephen Eilert (Chief Operating Officer) and Kathleen St. Jean (Chief Commercial Officer); and Petra Ottens and Susanne Veldhuis for their help with biochemical analyses. Figure 1 was created using Biorender.com.

## Supplementary Material


