# Supplementary material for: Perfusate Biomarker Comparison During Renal Hypothermic and Normothermic Machine Perfusion: Do These Techniques Provide Similar Insights?
Source: Transplantation. 2025 Jun 9;109(10):e554–66. doi: 10.1097/TP.0000000000005440 (PMC12453108; doi:10.1097/TP.0000000000005440)
Supplement: Supplementary file 1 [file tpa-109-e554-s001.pdf]

Table S1

Perfusate constituents	*Porcine*	*Human*
**Constituent**	**Volume (mL)**	**Volume (mL)**
**Total volume (mL)**	1195	1380
**(Autologous) red blood cells**	404	336
**Human albumin 200 g/L**<br>(Sanquin Plasma Products B.V., Amsterdam, the Netherlands)	180	200
**Sodium chloride 0.9%**<br>(Fresenius Kabi Nederland B.V., Zeist, the Netherlands)	451	500
**Sodium bicarbonate 8.4%**<br>(B. Braun Melsungen AG, Melsungen, Germany)	27	40
**Calcium gluconate 10%**<br>(B. Braun)	12	8
**Mannitol 15%**<br>(Baxter B.V., Utrecht, the Netherlands)	12	10
**Amoxicillin-clavulanate 1200 mg**<br>(Sandoz B.V., Almere, the Netherlands) **dissolved in 20 mL sterile water** (Fresenius Kabi)	16	n/a
**Cefazolin 1 g**<br>(Vianex SA, Patra, Greece) **dissolved in 20 mL sterile water** (Fresenius Kabi)	n/a	20
**Aminoplasmal 10%**<br>(B. Braun)	n/a	10
**Sodium phosphate 3 mmol/mL**<br>(Apotheek A15, Gorinchem, the Netherlands)	0.24	0.3
**Glucose 5%**<br>**(**Baxter B.V.)	13.5	28
**Magnesium sulfate 100 mg/mL**<br>(Teva Nederland B.V., Haarlem, the Netherlands)	1.2	1
**Potassium chloride 1 mmol/mL**<br>(Centrafarm B.V., Etten-Leur, the Netherlands)	1.2	n/a
**Heparin** (10000 U/mL)<br>(LEO Pharma B.V., Amsterdam, the Netherlands)	n/a	0.25
**Sterile water**<br>(Fresenius Kabi)	90	227
**Creatinine**<br>(Sigma-Aldrich, Zwijndrecht, the Netherlands)	95.7 mg	115.2 mg
**Cernevit** (Baxter B.V.) **dissolved in 5 mL sodium chloride 0.9%** (Fresenius Kabi)	n/a	1
**Verapamil 2.5 mg/mL**<br>**(Centrafarm B.V.)**	n/a	1
**Infusion solution constituents**		
**Infusion rate (mL/h)**	13.3	3
**Aminoplasmal 10%**<br>(B. Braun)	55.4	n/a
**Cernevit** (Baxter B.V.) **dissolved in 5 mL sodium chloride 0.9%** (Fresenius Kabi)	2	n/a
**Insulin 100 IU/mL**<br>**(Novo Nordisk A/S, Bagsværd, Denmark)**	0.4	n/a
**Glucose 5%**<br>(Baxter B.V.)	36.3	n/a
**Verapamil 2.5 mg/mL**<br>**(Centrafarm B.V.)**	2.3	4
**Sodium chloride 0.9%**<br>(Fresenius Kabi)	n/a	36
n/a: not applicable		

SDC, Materials and Methods

During $HMPO_2$ of porcine kidneys, perfusate samples were taken after 15 min of perfusion and after 6 hours. Samples were collected at identical time points during NMP to facilitate a direct comparison with the $HMPO_2$ samples. In the discarded human kidney cohort, $HMPO_2$ perfusate samples were obtained from the circuit after 15 min, 2 hours, and 4 hours of perfusion and samples were collected at the same time points during NMP. In the $HMPO_2$ and NMP perfusate samples of the porcine and discarded human kidney perfusions concentrations of aspartate aminotransferase (ASAT), lactate dehydrogenase (LDH), N-acetyl-β-glucosaminidase (NAG), and tissue inhibitor of metalloproteinases-2 (TIMP-2) were determined. In addition, heart-type fatty acid binding protein (H-FABP) was measured in the perfusate of the discarded human kidney perfusions. ASAT and LDH measurements were performed at the clinical laboratory of our hospital. NAG activity was measured with a colorimetric assay with p-nitrophenyl-N-acetyl-β-glucosaminide as a substrate at a pH of 4.25. TIMP-2 was measured using a commercially available enzyme-linked immunosorbent assay (ELISA) (Thermo Fisher Scientific, Waltman, MA, USA). H-FABP was quantified using a commercially available human H-FABP ELISA (R&D Systems, Minneapolis, MN, USA).

Due to the differences in circulating volume between $HMPO_2$ and NMP, the measured biomarker concentration was adjusted for volume to derive the absolute amount of biomarker produced per 100 grams of renal tissue based on renal weight prior to NMP.

Table S2

Biomarker	Minimal WI<br>*Median + IQR*	75 min WI<br>*Median + IQR*	*p* value
**ASAT (U/100g)**			
$HMPO_2$ 15 min	0.61 (0.55 – 0.69)	0.99 (0.67 – 1.29	**< 0.001**
$HMPO_2$ 360 min	2.46 (1.78 – 3.08)	6.23 (4.92 – 8.58)	**< 0.001**
NMP 15 min	11.75 (8.03 – 15.94)	20.71 (14.4 – 30.21)	**0.001**
NMP 360 min	33.19 (25.57 – 41.07)	119.8 (96.81 – 135.6)	**< 0.001**
**LDH (U/100g)**			
$HMPO_2$ 15 min	2.53 (1.94 – 2.88)	4.35 (3.43 – 5.8)	**0.001**
$HMPO_2$ 360 min	17.52 (13.46 – 20.8)	23.36 (19.26 – 34.39)	**< 0.001**
NMP 15 min	36.87 (28.26 – 47.23)	42.56 (33.12 – 52.19)	0.273
NMP 360 min	108.3 (88.17 – 132.5)	157.5 (27.8 – 186.8)	**< 0.001**
**NAG**			
$HMPO_2$ 15 min	1.80 (1.61 – 1.97)	1.76 (1.59 – 1.90)	0.126
$HMPO_2$ 360 min	1.72 (1.50 – 1.92)	1.77 (1.58 – 1.88)	0.316
NMP 15 min	3.26 (2.87 – 5.15)	3.27 (2.81 – 4.71)	0.321
NMP 360 min	12.59 (7.7 – 18.22)	9.80 (7.39 – 12.52)	**0.020**
**TIMP-2**			
$HMPO_2$ 15 min	161.8 (111.2 – 193.3)	233.1 (174.4 – 265.6)	**< 0.001**
$HMPO_2$ 360 min	344.2 (304.9 – 395.2)	424.3 (340 – 491.5)	**< 0.001**
NMP 15 min	499.6 (403.8 – 562.6)	675.9 (589.2 – 808.9)	**< 0.001**
NMP 360 min	1593 (1429 – 1785)	1872 (1706 – 1985)	**< 0.001**

ASAT, aspartate aminotransferase; $HMPO_2$, oxygenated hypothermic machine perfusion; LDH, lactate dehydrogenase; NAG, N-acetyl-b-glucosaminidase; NMP, normothermic machine perfusion; TIMP-2, tissue inhibitor of metalloproteinases 2.
